# From tokenism to youth leadership in child and adolescent mental health research

**DOI:** 10.3389/phrs.2026.1609722

**Published:** 2026-05-26

**Authors:** Sophie Mae Harrington, Paul Louis Fiedler, Katerina Sidiroglou, Nadia Ahmed, Hannah Brunskill, Jennifer Hall, Ledia Lazëri, João Breda

**Affiliations:** 1 World Health Organization Office on Quality of Care and Patient Safety, Athens, Greece; 2 World Health Organization Regional Office for Europe, Copenhagen, Denmark

**Keywords:** child and adolescent, leadership, mental health, youth participation, youth participatory research

## Introduction

Strengthening the quality of child and youth mental healthcare (CAYMH) is a priority across the WHO European Region [1]. One core element of this is to enhance data collection and research [2]. There is increasing evidence that inputs from people with lived experience improve the quality and relevance of research [3]. Youth engagement in the research process for CAYMH is becoming increasingly common with varying levels of involvement. According to Hart’s [4] ladder of participation, involvement can range from ‘non participation’ (i.e., manipulative, decorative, or tokenistic) to varying degrees of ‘participation’ (including assigned but informed, consulted and informed), with child or youth-led participation with shared decision-making power representing the highest level.

In this commentary, we define “youth leadership” as this highest level of participation; participation which allows decision-making to be done by those most affected by outcomes across project phases. Youth leadership has demonstrated positive outcomes for young people, including empowerment, emotional regulation, self-efficacy, and skill development [5, 6].

Despite these benefits, many participatory approaches for youth participation in CAYMH research remain tokenistic, lacking real decision-making power. The authors of this commentary have drawn on their collective youth lived (SMH, PF, AS, NA) and professional (HB, JH, LL, JB) expertise to highlight challenges related to youth participation and ‘lived experience’. We urge institutions to go beyond “tokenistic” youth advisory roles to embed youth leadership with formal decision-making authority, thereby improving research quality, ethical norms, and legitimacy.

## Organisational barriers to youth leadership

There are many challenges for implementing youth leadership from the organisational perspective. Youth leadership can be viewed as too risky, time-consuming, or complex, particularly within mental health contexts where safeguarding is a priority [7]. Additional barriers include legal challenges of including minors, unclear roles, inadequate resourcing, hierarchical decision-making, a lack of clear institutional guidance, or organisational norms that do not yet view youth leadership as legitimate or influential [7, 8].

While these concerns are legitimate, we argue that there are ways to overcome these challenges. Funders and donors can ensure that youth leadership is a prerequisite for approving any projects. Government decision-makers can integrate youth leadership in policy and guideline documents, enabling youth voices to shape decisions at all levels. Organisations can include funds for youth leadership in all budgets related to CAYMH. Budgeting considerations include youth leadership roles with real authority and equitable decision-making power, governance structures that include youth membership, capacity-building for all the organisation on how to implement safe youth leadership, fair remuneration for youth leaders, developing safeguarding policies, and supervisory structures. When creating opportunities for participation, we call for institutions to recognise lived experience as expertise, and explicitly define what level of participation they are seeking.

## Defining “lived experience”

Mental health exists across a spectrum and varies across an individual’s life, and is not something someone “has” or “doesn’t have.” A challenge is how “lived experience” is defined, and who is perceived as credible. “Lived experience” is inconsistently defined across settings, with informal norms shaping which kinds of contributions are valued. Drawing on our experience in youth participation initiatives, legitimacy within these spaces is often implicitly linked to a willingness to disclose personal or emotionally salient experiences. The absence of a formal diagnosis is sometimes interpreted as a lack of credibility or authenticity, or further treated as an exclusionary criterion, suggesting that only institutionally approved youth can participate. This can place young people under pressure to disclose more than they are comfortable with in an attempt to validate their own experiences. Over time, this narrows participation to those willing to disclose personal or sensationalist narratives, marginalising other forms of knowledge and expertise.

To overcome this issue, we call on organisations to first consider what kind of lived experience expertise is required for the work, and to be clear about this from the outset. Lived experience expertise encompasses a broad range of qualities, including culture, insight, knowledge, skills, and identity. Being clear about these boundaries from the outset empowers young people to independently make a decision about whether they would fit the criteria and are willing to partake.

## Disclosure and consent

To implement youth leadership, young people must feel safe to participate and hold decision-making power. A significant barrier has been unclear expectations around disclosure and how it is handled. In our collective experience, young people are often inadequately informed what their participation entails or how their disclosures will be used. When contributions or personal disclosures are mishandled, such as sharing them without explicit consent, it can leave young people feeling unnecessarily exposed and vulnerable.

Young people must always be offered anonymity when sharing their input, and explicitly consent to sharing this input before any unfiltered contributions can be used. This requires consent to be an ongoing, revisable process rather than a one-time procedural requirement, particularly concerning the use, circulation, and interpretation of personal narratives. We also call for the dissemination of contributions to include young people, and to focus on actionable insights (e.g., what needs to change) rather than sensationalist individual experiences. To empower young people as leaders in the process, organisations should create youth leadership opportunities at every stage, not as procedural compliance, but as a genuine transfer of decision-making power.

## Youth leadership embedded in every step

Often, young people’s inputs are confined to providing insights on isolated aspects, which limits their ability to provide meaningful input and reinforces power imbalances. When institutions fail to prioritise transparency, full contextual understanding, and ethical engagement, they perpetuate a cycle of tokenism that hinders the transformative potential for authentic youth leadership.

To overcome this, institutions should be transparent about their own knowledge and experience with youth involvement and co-production, including whether they have dedicated staff with expertise to facilitate youth leadership safely and ethically. They should also provide full transparency of the entire research process. An accessible document that clearly outlines each stage of the process, including the specific aspects young people are being asked to contribute to, and how their contributions will be used, can help achieve this.

### Conclusion

We call for a fundamental shift of youth participation in CAYMH research: from tokenistic participation to genuine youth leadership, where young people hold real decision-making power. The actions outlined in this commentary aim to overcome some of the common challenges for youth leadership. With system level change, commitment, and adequate resourcing, organisations can be enabled to embed youth leadership through all work on CAYMH.

Youth leadership strengthens research by grounding it in lived experience; it allows policies to be based on research that reflects young people’s needs, and upholds the ethical principle that those most affected by decisions have a meaningful role in shaping them. Central to this shift is recognising lived experience as a legitimate, diverse, and multifaceted expertise that is not defined by diagnosis or disclosure.

We therefore call on funders, ethics committees, policymakers, and research institutions to mandate and resource youth leadership across all phases of CAYMH research. Embedding youth leadership is more than a procedural improvement. It is an investment in better science, fairer systems, and mental health policies that truly serve young people.
